# Supplementary material for: Content-rich biological network constructed by mining PubMed abstracts
Source: BMC Bioinformatics. 2004 Oct 8;5:147. doi: 10.1186/1471-2105-5-147 (PMC528731; doi:10.1186/1471-2105-5-147)
Supplement: Additional File 5 — The original Chilibot query results of the term "long-term potentiation (LTP)" and 22 other terms, limiting the latest references analyzed to the years 1990, 1995, 2000, and 2004. [file 1471-2105-5-147-S5.bz2 › chilibotAdditionalFile5/ltp2004/html/ERK_TAU.html]

 


 **ERK** and **TAU** 
  
Found 34 abstracts in PubMed,  **30 abstracts were retrieved and analyzed**.  


---

 Search Google  |
 PDF files only 
|  EDU domain only 

---

**Interactive relationship** (e.g. stimulation, inhibition, etc)

- Hyperphosphorylation of  **tau**  is mediated by  **ERK**  activation during anticancer drug induced apoptosis in neuroblastoma cells.  Ref: 11170175 J Neurosci Res, 2001
- The  **ERK**  and JNK cascades, which are downstream of Ras, have opposing effects on  **tau**  promoter activity  **ERK**  induces  **tau**  promoter activity, JNK inhibits it.  Ref: 9422391 J Neurochem, 1998
- We next showed that a sustained activation of  **ERK**  extracellular signal regulated kinase induced both  **tau**  phosphorylation and apoptosis during paclitaxel treatment 1 microM.  Ref: 11170175 J Neurosci Res, 2001
- The mitogen activated protein kinases MAPKs  **ERK**  and JNK were activated by step  **tau**  but not by ramping to the same  **tau**  level.  Ref: 11744620 FASEB J, 2002
- Increased expression of the phosphorylated forms of MAPK  **ERK** , SAPK JNK, p38 and GSK 3beta was found in grains and  **tau**  containing cells in AGD.  Ref: 12580546 Brain Pathol, 2003
- The abnormal hyperphosphorylation of  **tau**  in Alzheimer s disease AD has been proposed to involve the extracellular signal regulated protein kinase  **ERK**  of the mitogen activated protein MAP kinase family.  Ref: 12531514 Brain Res Mol Brain Res, 2002
- **Tau**  promoter activity in PC12 cells is correlated with a short term activation of  **ERK** , which declines after a few hours and is followed by an activation of the inhibitory JNK cascade 76 h later.  Ref: 9422391 J Neurochem, 1998
- Abeta 1 42 induced JNK and  **ERK**  activation in rabbit hippocampus is differentially regulated by lithium but is not involved in the phosphorylation of  **tau** .  Ref: 14625087 Brain Res Mol Brain Res, 2003
- These results demonstrate increased phosphorylated active  **ERK**  expression in association with early  **tau**  deposition in neurones and glial cells in taupathies, and suggest activated Ras as the upstream activator of the MEK  **ERK**  pathway of  **tau**  phosphorylation in AD.  Ref: 11303790 Brain Pathol, 2001
- 0.05 activation of the Raf 1 MEK 1  **ERK**  1 2 pathway, which was obligatory for the expression of COX 2 and secretion of PGF but was not altered by cotreatment with rbIFN  **tau** .  Ref: 11207214 Biol Reprod, 2001
- These observations suggest that the induction and inhibition of  **tau**  promoter are mediated by alternate  **ERK**  and JNK activities, which may underlie a mechanism to turn on and off genes during PC12 cell differentiation.  Ref: 9422391 J Neurochem, 1998
- Single and double labeling immunohistochemistry to MAPK  **ERK**  P and p38 P disclosed that MAPK  **ERK**  P appeared at early stages of  **tau**  phosphorylation in neurons and glial cells in tauopathies,.  Ref: 11810404 J Neural Transm, 2001
- In vitro, these  **tau**  phospho epitopes can be phosphorylated by the kinases  **ERK**  and JNK, and the kinases themselves are negatively regulated by PP2A.  Ref: 12937125 Am J Pathol, 2003
- MAPK  **ERK**  P was found in a subset of neurons and glial cells bearing abnormal  **tau**  deposition, but rarely in neurofibrillary tangles.  Ref: 11810404 J Neural Transm, 2001
- These observations, together with previous results of in vitro studies, support the idea that several MAPK  **ERK** , SAPK JNK, p38 and CaM kinase II may participate in  **tau**  phosphorylation in tauopathies.  Ref: 11810404 J Neural Transm, 2001
- MAPK  **ERK**  P and p38 P co localize only in a subset of neurons and glial cells with phosphorylated  **tau**  deposits.  Ref: 11810404 J Neural Transm, 2001
- Between 80% and 90% of IBM vacuolated muscle fibers contained well defined  **ERK**  immunoreactive inclusions, which were co localized by light microscopy, with phosphorylated  **tau**  in 70 to 80% of those fibers.  Ref: 10854206 Am J Pathol, 2000
- Finally, during a column fractionation and purification of  **Erk**  kinases from rat brain extracts, fractions enriched in  **Erk**  kinase activity also exhibited co elution of phosphorylated NF H, tubulin,  **tau**  and cdk5.  Ref: 10762698 Brain Res Mol Brain Res, 2000
- These findings indicate that MAPK  **ERK**  P, SAPK JNK P and p 38 P are differentially expressed in association with  **tau**  deposits in tauopathies.  Ref: 11810404 J Neural Transm, 2001
- MAPK  **ERK**  P, SAPK JNK P, p38 P and GSK 3beta P were expressed in  **tau**  containing astrocytes and in oligodendrocytes with coiled bodies.  Ref: 12580546 Brain Pathol, 2003
- MAPK  **ERK**  P, SAPK JNK P, p38 P and GSK 3beta P were also expressed in  **tau**  containing astrocytes and in oligodendrocytes with coiled bodies.  Ref: 12581337 Neuropathol Appl Neurobiol, 2003
- Treatment with 7 mM lithium inhibits apoptosis, modulates JNK and  **ERK**  and does not affect the phosphorylation of  **tau** .  Ref: 14625087 Brain Res Mol Brain Res, 2003

**Parallel relationship** (e.g. studied together, co-existance, homology, etc.)

- Our findings imply that PP2A is a negative regulator of the  **ERK**  and JNK signaling pathways in vivo, suggesting that in AD,  **tau**  hyperphosphorylation may be caused in part by PP2A dysfunction.  Ref: 12937125 Am J Pathol, 2003
- **ERK**  is able to phosphorylate  **tau**  protein, so the up regulation of mGluR2 in vulnerable neurons may represent the upstream mediator of abnormal  **tau**  phosphorylation in AD.  Ref: 14872255 Acta Neuropathol (Berl)Acta Neuropathol (Berl), 2003
- In this model, the intracellular amyloid beta phenotype is accompanied by increased MAPK  **ERK**  activity and  **tau**  hyperphosphorylation.  Ref: 12428762 Mol NeurobiolMol Neurobiol, 2003
- 0.001, indicating a link between  **ERK**  activation,  **tau**  phosphorylation and apoptosis.  Ref: 11170175 J Neurosci Res, 2001
- The inhibition of  **ERK**  activation by using the pharmacological MEK1 2 inhibitor, PD98059 50 microM, or an antisense strategy, reduced  **tau**  phosphorylation and neuronal apoptosis P <.  Ref: 11170175 J Neurosci Res, 2001
- In conclusion, COX 2 expression and PGF secretion induced by PDBu is mediated through the Raf 1 MEK 1  **ERK**  1 2 pathway, but this pathway is not disrupted by rbIFN  **tau** .  Ref: 11207214 Biol Reprod, 2001
- However, the H2O2 treated cells also manifested increased expression of growth and sprouting molecules, including GAP 43, nitric oxide synthase 3, neuronal thread protein, proliferating cell nuclear antigen, and phospho  **Erk**  MAPK, and normal levels of the AD associated ~41 kD NTP species, cyclin dependent kinase 5, and phospho  **tau** .  Ref: 12214088 J Alzheimers Dis< MedlineTA>J Alzheimers Dis, 2001
- Further, compound SB216763 inhibited  **Tau**  phosphorylation at Ser396 and stabilized cytosolic beta catenin, consistent with the inhibition of glycogen synthase kinase 3 beta GSK 3 beta, but failed to reproduce lithium effects on MEK and  **ERK**  phosphorylation and cell cycle arrest.  Ref: 14511119 J Neurochem, 2003
- Our study suggests 1 that  **ERK** , a signal transducer, might play a role in IBM pathogenesis, including participation in the pathological phosphorylation of IBM  **tau** .  Ref: 10854206 Am J Pathol, 2000
- Furthermore, activation of A1R led to and  **ERK**  dependent increase of  **tau**  phosphorylation and translocation towards the cytoskeleton.  Ref: 14655750 Brain Pathol, 2003
- Previous studies have shown active Ras and increased mitogen activated protein kinase MAPK  **ERK**  expression in neurones and glial cells with abnormal  **tau**  deposition in AD and other tauopathies.  Ref: 11679086 Neuropathol Appl Neurobiol, 2001
- The present study examines the involvement of the Ras MEK  **ERK**  pathway of  **tau**  phosphorylation in Alzheimer disease AD, Pick s disease PiD, progressive supranuclear palsy PSP and corticobasal degeneration CBD, by Western blotting, single and double labelling immunohistochemistry, and p21Ras activation assay.  Ref: 11303790 Brain Pathol, 2001
- Phosphorylated mitogen activated protein kinase MAPK  **ERK**  P, protein kinase of 38 kDa p38 P, stress activated protein kinase SAPK JNK P, and calcium calmodulin dependent kinase II CaM kinase II are differentially expressed in  **tau**  deposits in neurons and glial cells in tauopathies.  Ref: 11810404 J Neural Transm, 2001
- The immunoreactivity of cortical and brainstem type Lewy bodies has been investigates with antibodies to the cyclin dependent kinase 5 cdk5, to the extracellular regulated kinase 1  **ERK**  1, and to the cdc2p34 kinase and with antibodies specific for phosphorylation epitopes typical of paired helical filament  **tau**  PHF  **tau** .  Ref: 7485409 Am J Pathol, 1995
- Phospho specific  **tau**  antibodies Thr181, Ser202, Ser214, Ser396 and Ser422, and antibodies to glycogen synthase kinase 3alpha beta GSK 3alpha beta and to phosphorylated P mitogen activated protein kinase extracellular signal regulated kinases MAPK  **ERK** , stress activated protein kinase c Jun N terminal kinase SAPK JNK, p38 kinase p38 and GSK 3betaSer9 have been used to gain understanding of the identification of phosphorylation sites, as well as of the specific kinases that regulate  **tau**  phosphorylation at those specific sites, in a familial tauopathy.  Ref: 12581337 Neuropathol Appl Neurobiol, 2003
- Doxorubicin 0.2 microM, an anticancer drug whose mechanism of action is independent of microtubules, also induced  **ERK**  activation,  **tau**  phosphorylation and apoptosis.  Ref: 11170175 J Neurosci Res, 2001
- A possible link in support of this hypothesis is that one of the most striking features of AD, the abnormal accumulation of highly phosphorylated  **tau**  and neurofilament proteins, may be brought about by extracellular receptor kinase  **ERK**  whose activation is a common response to oxidative stress.  Ref: 10439473 Neuroreport, 1999
- Stress induced  **tau**  phosphorylation in mouse strains with different brain  **Erk**  1 2 immunoreactivity.  Ref: 9821159 Neurochem Res, 1998
- Both cortical and brainstem type Lewy bodies in diffuse Lewy body disease and brainstem type Lewy bodies in Parkinson s disease were found to be immunoreactive for cdk5 but not for cdc2p34 or  **ERK**  1 or with the PHF  **tau**  antibodies.  Ref: 7485409 Am J Pathol, 1995
